# Creating symbolic cultures of consumption: an analysis of the content of sports wagering advertisements in Australia

**DOI:** 10.1186/s12889-016-2849-8

**Published:** 2016-03-01

**Authors:** Emily G. Deans, Samantha L. Thomas, Mike Daube, Jeffrey Derevensky, Ross Gordon

**Affiliations:** Centre for Population Health Research, School of Health and Social Development, Faculty of Health, Deakin University, Melbourne, Australia; Public Health Advocacy Institute of Western Australia, Curtin University, Perth, Australia; International Centre for Youth Gambling Problems and High Risk Behaviours, McGill University, Montreal, Canada; Department of Marketing and Management, Faculty of Business and Economics, Macquarie University, Sydney, Australia

**Keywords:** Gambling, Advertising, Sport, Marketing, Content analysis, Men

## Abstract

**Background:**

Since 2008, Australia has seen the rapid emergence of marketing for online and mobile sports wagering. Previous research from other areas of public health, such as tobacco and alcohol, has identified the range of appeal strategies these industries used to align their products with culturally valued symbols. However, there is very limited research that has investigated the tactics the sports wagering industry uses within marketing to influence the consumption of its products and services.

**Method:**

This study consisted of a mixed method interpretive content analysis of 85 sports wagering advertisements from 11 Australian and multinational wagering companies. Advertisements were identified via internet searches and industry websites. A coding framework was applied to investigate the extent and nature of symbolic appeal strategies within advertisements.

**Results:**

Ten major appeal strategies emerged from this analysis. These included sports fan rituals and behaviours; mateship; gender stereotypes; winning; social status; adventure, thrill and risk; happiness; sexualised imagery; power and control; and patriotism. Symbols relating to sports fan rituals and behaviours, and mateship, were the most common strategies used within the advertisements.

**Discussion/Conclusions:**

This research suggests that the appeal strategies used by the sports wagering industry are similar to those strategies adopted by other unhealthy commodity industries. With respect to gambling, analysis revealed that strategies are clearly targeted to young male sports fans. Researchers and public health practitioners should seek to better understand the impact of marketing on the normalisation of sports wagering for this audience segment, and implement strategies to prevent gambling harm.

## Introduction

### Background

Each year, about 400,000 Australian adults experience gambling related problems or are at moderate risk of experiencing problems [1]. Whilst gambling is promoted by the gambling industry as an enjoyable leisure activity [2], there is significant evidence that gambling results in a range of health and social harms to individuals, their families and communities [3, 4]. Specific harms include anxiety and depression [5], lowered work productivity and job loss [6], suicidal ideation and attempts [7], substance abuse [8], family and partner breakdown [9], financial hardship [10], crime [11], homelessness [12] and, domestic violence [13, 14].

While Electronic Gambling Machines (pokies or slots) have been identified as the most harmful form of gambling [15–17], there is limited information about the impacts of other forms of gambling, such as sports wagering, on the health and wellbeing of individuals and communities. This includes the range of strategies that may be used by the wagering industry to promote their products and services [18, 19]. Sports wagering is a growing segment of the gambling market in Australia. According to recent prevalence data from the state of Victoria, 4.82 % of the total adult population, 10.56 % of 18–24 year olds, and 8.25 % of 25–34 year olds participated in sports and event betting in 2014 [20]. In 2014, it was also estimated that Australians would gamble an average $1200 per person on 90,000 different races, soccer matches, the National Rugby League premiership, basketball games, and other national and international sporting events [21].

Researchers have suggested that there are a number of factors that may stimulate the uptake of sports wagering products, as well as the risks associated with these products. First is the accessibility of wagering opportunities, with most services now available online. By 2014, it was estimated that 50 % of all wagering industry profits were expected to stem from online channels, a figure anticipated to rise to 70 % by 2023 [22]. The ubiquitous nature of mobile phone devices has revolutionized sports wagering opportunities, allowing greater ease of access 24 h per day, 7 days per week. Second is the way in which sports wagering is marketed in Australia, including the alignment of sports wagering marketing with Australian sporting teams, codes, and games. Some researchers have argued that it is the proliferation of marketing for sports wagering products and services that has had the greatest influence in stimulating the consumption of sports wagering products and services [23–26]. While researchers have documented the extent and nature of sports wagering marketing activities during sporting matches, there is limited understanding of the range of appeal strategies that are used within sports wagering advertising campaigns.

### Advertising and symbolic consumption

It is clear that marketing is used by industries to influence the social acceptance of products, and rework the cultural meanings that individuals assign to these products [27, 28]. Advertising frequently plays a pivotal role in shaping an individual’s beliefs about a particular product, and their intentions to use that product [29–33]. It is important to note that this is not a ‘one way’ process. Consumers also play a role in co-creating the meanings that they attach to particular products [34]. This process is known as ‘*symbolic consumption’,* whereby consumers use the social information attached to products (initially created via marketing tactics) to shape their own self-image [35]. Relationships are subsequently formed with these products (and their distinct brands), and the behaviours that are associated with the consumption of these products are used to create both individual and group identities [36]. Researchers suggest that young adults are particularly influenced by the symbolic properties of products, which are used to construct social identities [37, 38]. Thus, consumer goods and services often have a significance that goes beyond their basic functional value [27–29].

Symbolic consumption processes have underpinned the promotion of many products that are potentially harmful for individuals and communities. For example, researchers identified that symbolic consumption (and identity creation) were at the forefront of marketing efforts used by the tobacco and alcohol industries. Tobacco industry marketing overlaid cigarettes with marketing tactics that emphasised values such as social status and acceptance, glamour, and adventure to sell their products [39–41]. The appeal strategies adopted by the alcohol industry are similar, but are particularly recognised for associating alcohol with ideals of enhanced masculinity and social worth [42–44].

What is less clear is how the Australian sports wagering industry uses symbolic consumption processes to align its products with socially and culturally valued meanings. In turn, how might these processes highlight the audience segments that have the most potential to be influenced by these gambling marketing practices? Existing descriptive studies on the content of gambling marketing more broadly have shown similar trends to those seen in alcohol and tobacco marketing, with advertising emphasising glamour and excitement, and exaggerating the associated social benefits of the uptake of gambling practices [45–48]. Researchers specifically examining the marketing of sports wagering have suggested that positioning with sport provides the wagering industry with an immediate alignment with an activity that is both socially and culturally valued in communities [23, 24, 26, 49–51]. A recent study examining the social media marketing practices of the sports wagering industry identified the strong links being established between gambling brands and sporting organisations and codes [18]. Promotional messages on social media, for example, often used real-time game related hashtags, and engaged in commentary related to the live sports match [18].

This study aimed to provide both a theoretical and empirical understanding of the use of symbolic appeal strategies in sports wagering advertising in Australia. The study is exploratory and interpretative, while developing and applying a coding template that may be used by other researchers to: a) monitor symbolism in sports wagering over time, and b) to map marketing strategies between companies. We conclude by discussing the role that symbolic consumption may play in normalising sports wagering as an individual and group level activity.

## Methods

A mixed methods interpretive content analysis was used to explore the written, verbal and visual symbolism used within a sample of 85 sports wagering advertisements [52]. An interpretative content analysis differs from traditional content analyses in the way in which data is coded [53]. Researchers adopting this methodology code the data with consideration of the rest of the text, and the latent content and meaning [53].

### Sample selection

The sample chosen for this study did not aim to include and analyse all sports wagering advertisements in Australia. To collect such a sample would require the purchase of expensive commercial monitoring data which was beyond the financial scope of this study. Rather, we used a comprehensive internet search to identify a range of advertisements with diverse creative strategies, from a range of wagering providers. Internet searches are becoming an increasingly common method used by researchers to identify commercial marketing data [18]. We provide a step-by-step description of the process used to identify the advertisements for this study.

### Step One: identifying a sampling time frame

For this study we decided to focus on advertisements for sports wagering products played in Australia between January 2008 and October 2015. We chose 2008 as a starting date as this was when a High Court ruling (in a case between the state of Western Australia (WA) and Betfair Pty Ltd) invalidated laws in WA which were in place to prohibit licensed betting exchanges offering markets on WA races, and to prohibit WA residents from using licensed betting exchanges [54]. The increase in marketing for sports wagering products has in part been attributed to this ruling as it prompted strong interest in the Australian betting market from international wagering operators [19, 23]. Campaigns in our sample may have been developed as early as 2008 and as late as 2015. During this time period, campaign strategies certainly would have evolved and changed. However, we were unable to ascertain television airing time as this information was unavailable when the advertisements were identified.

### Step Two: identifying wagering brands

We aimed to include major Australian wagering brands that had been advertised from 2008 to 2015. To identify these brands we referred to previous research that had documented the presence of sports wagering marketing (including brands) during sporting matches [23, 26]. We also crosschecked the identified brands with another study exploring sports wagering marketing on social media [18]. The 11 sports wagering brands included were: Bet365.com.au; Betfair.com.au; Centrebet.com.au; CrownBet.com.au; Ladbrokes.com.au; Sportingbet.com.au; Sportsbet.com.au; TAB.com.au; TomWaterhouse.com; UBET.com.au; and Williamhill.com.au. Very few of the chosen brands had advertised across the complete 2008–2015 time period. The sample, therefore, included brands that no longer exist in the Australian market (e.g. Centrebet.com.au and Sportingbet.com.au, were acquired by British parent company William Hill [55]), and newer brands that had entered the Australian market during the last 1–3 years (e.g. Crownbet.com.au and Ladbrokes.com.au).

### Step three: internet search strategy

Advertisements were identified using two main strategies: 1) By conducting broad internet searches (Google searches) using sports wagering company brand names along with the terms ‘advertisement’, ‘TV ad’, ‘promotion’, and ‘marketing’; 2) Using specific searches for advertisements on gambling operators websites, YouTube channels, and marketing trade websites. For example, many wagering companies have dedicated ‘channels’ on YouTube where advertisements are uploaded (along with other related content) [18]. It is important to note that without accessing commercial monitoring data, we cannot be 100 % certain that all of the advertisements sampled were played on Australian television. Furthermore, one of the methodological difficulties with using YouTube as a data collection repository is that the date stamp may not accurately reflect when/if the advertisement was played on Australian television.

### Step four: inclusion/exclusion criteria

To be included in the sample, advertisements had to contain a message about ‘responsible gambling’ or ‘gamblers’ help services’. This is a regulatory requirement for all sports wagering advertisements played on Australian television [56]. Advertisements that had a YouTube date stamp between January 2008 and October 2015 were included, while advertisements from sports wagering companies that promoted non-monetary competitions (such as sports tipping competitions) were excluded. We also excluded ‘market update’ advertisements, which lacked a story line and only contained updated odds information. This is because we were interested in the broader creatives associated with gambling advertisements rather than merely informational advertising.

A total of 85 advertisements were identified and included in the sample. These included Bet365.com.au (*n* = 2), Betfair.com.au (*n* = 6), Centrebet.com.au (*n* = 10), CrownBet.com.au (*n* = 5), Ladbrokes.com.au (*n* = 9), Sportingbet.com.au (*n* = 9), Sportsbet.com.au (*n* = 18), TAB.com.au (*n* = 12), TomWaterhouse.com (*n* = 4), UBET.com.au (*n* = 4, and Williamhill.com.au (*n* = 6). The inclusion of a range of old and new brands within the sampling timeframe, and a reliance on advertisements that were uploaded to the internet, may in part explain why there was some variety in the number of advertisements identified for each brand.

### Development of coding template

We used directed and inductive approaches to develop a coding template to analyse the advertisements [57]. The directed approach involved reviewing published tobacco, alcohol and gambling research that described specific thematic appeals within marketing for these products [40, 43, 44, 48]. From this review, we documented a wide variety of strategies that had been identified within previous research. We then grouped these smaller themes into seven broad coding categories – Adventure, Thrill and Risk; Gender Stereotypes; Mateship and Peer Bonding; Patriotism; Sexualised Imagery; Social Status; and Winning. We then created coding definitions for each of these categories which were used to guide the interpretation of the advertisements (Table 1).

**Table 1:** Coding framework

Theme/ Symbol	Generic definition	Applicability to study of sports wagering advertisements
Themes based on existing literature		
Adventure, Thrill and Risk	Appeals that centre on adventure, freedom, thrill and risk taking activities [44].	Sports wagering is an activity designed for those who love an adrenaline rush, who take risks, and who are adventurous.
Gender Stereotypes	Appeals that reinforce hegemonic masculinity or sex roles [42–44, 62].	Makes reference to the gender stereotypical male, and promotes that such ideals can be achieved via product consumption, e.g. strength, boldness, muscled, driven, handsome, strong, stoic, powerful (power relations between men/women) - ‘real men gamble.’
Mateship and Peer Bonding	Appeals centred upon peer belonging and social bonding, e.g. The depiction of mates in humorous scenarios together/ ‘friendly belittlement’ of one’s mate [58].	Sports wagering brings ‘mates’ together, and is an important element to peer group belonging, e.g. set in a pub, groups gathered enjoying a drink.
Patriotism	The concept of group loyalty extended to community and to country [44, 62].	Sports wagering is a great way to show pride in, support for, and loyalty towards one’s country.
Sexualised Imagery	Eroticized portrayals of women or men, including acts, gestures, clothing or hidden meanings, that accentuated sexual objectification [65].	Individuals who gamble are sexually successful, admired by, and confident with the opposite sex.
Social Status	Appeals centred upon improving or enhancing social status [48].	Individuals who gamble encompass what society considers successful –popular, wealthy, confident, attractive and influential.
Winning	The notion that winning money via gambling is easy and life-changing [46].	It is easy to feel accomplished (victorious) when wagering on sports, easy wins, and generous bonuses and rewards.
Themes developed from inductive analysis		
Happiness	Appeals of excitement, happiness and elation.	Wagering on sports makes you happy. Wagering on sports is fun.
Power and Control	Appeals of empowerment, superiority and control.	The portrayal that suggests individuals who gamble are powerful, wealthy and in control of their life. Additionally, implications that mobile wagering, coupled with increased betting options, aid in punting control.
Sports Fan Rituals and Behaviours	Sports fan behaviours as expressed by team colours and jerseys, crowd cheer, live matches, and sporting legends.	Sports wagering shows pride in, support for, and loyalty towards one’s sporting team. The fan experience is heightened when wagering is embedded in the established rituals of sports enthusiasts.

Because very few studies had investigated themes within sports wagering advertisements, we used an inductive approach to incorporate themes that were specifically relevant to sports wagering advertisements. To do this, Author One viewed each sports wagering advertisement that had been developed. The advertisements were viewed several times, were transcribed, and notes were taken about the content of each advertisement. We then used a thematic process of analysis to identify coding themes. Authors One and Two met to discuss the themes and to review the transcripts and descriptions of the advertisements. A selection of advertisements (with different themes and appeal strategies) were viewed and re-viewed by Authors One and Two, and themes were discussed. Broad themes were then reduced to a smaller number of categories which were then included in the final coding framework. These included Happiness; Power and Control; and Sports Fan Rituals and Behaviours (Table 1).

### Data analysis

To apply the coding framework each advertisement was viewed at least twice by Author One. A sample of 10 % of advertisements were then independently viewed and coded by another member of the research team. Similar to the process used in other interpretive content analyses [58], the coding between reviewers was then compared. Where there was disagreement, reviewers discussed these until a consensus was reached. Frequency counts were performed to quantify the major themes and associated symbols for each sports wagering advertisement. Using the transcribed and descriptive data about each advertisement, we then conducted a qualitative analysis of the appeal strategies [59]. This analysis aimed to provide narrative and illustrative information to supplement the quantitative data (a method used in other papers exploring the promotion of sports wagering during sporting matches) [23, 26]. We explored the key appeal strategies within each advertisement and compared and contrasted these themes across categories. Examples from this analysis are presented to illustrate key creative and plotline strategies.

## Results

The results of the quantitative analysis are presented in Table 2.

**Table 2:** Number of appeals by sports wagering brand

Symbol	Bet365	Betfair	Centre bet	Crown bet	Lad brokes	Sporting bet	Sports bet	TAB	Tom Water house	UBET	William Hill	Total
	(*n* = 2)	(*n* = 6)	(*n* = 10)	(*n* = 5)	(*n* = 9)	(*n* = 9)	(*n* = 18)	(*n* = 12)	(*n* = 4)	(*n* = 4)	(*n* = 6)	(*n* = 85)^a^
Sports Fan Rituals and Behaviours	1	5	10	5	5	9	9	8	4	4	6	66(78 %)
Mateship	0	0	3	0	9	4	12	7	1	4	1	41(48 %)
Gender Stereotypes	0	3	2	0	3	8	15	2	0	0	1	34(40 %)
Winning	0	3	0	5	5	0	5	5	2	2	3	30(35 %)
Social Status	0	2	3	1	4	0	4	3	4	1	3	25(29 %)
Adventure, Thrill and Risk	1	1	10	1	1	1	2	0	2	4	0	23(27 %)
Happiness	0	0	0	1	2	1	6	6	1	4	2	23(27 %)
Sexualised Imagery	0	2	5	1	2	1	5	4	1	0	0	21(25 %)
Power and Control	1	3	1	0	1	0	3	0	4	0	3	16(19 %)
Patriotism	2	2	0	0	0	0	0	4	1	3	0	12(14 %)

^a^Values do not add to 100 % because advertisements include more than one type of symbolic appeal

### Sports Fan rituals and behaviours

Over three quarters of advertisements utilised visual or verbal imagery relating to sports fan rituals and behaviours (*n* = 66, 78 %). Qualitative analyses revealed the way in which appeal themes specifically linked wagering with aspects of sports fans existing match experiences. This included imagery of fans at venues or watching games on television – cheering for their teams, waving banners, and wearing team uniforms, jerseys, or scarves. These advertisements were often linked to companies that had sponsorship deals with sporting teams or codes – in particular Centrebet.com.au (St Kilda Australian Football League (AFL) Football Club); TAB.com.au (‘Socceroos’- the Australian National Men’s Football (Soccer) Team); Williamhill.com.au (Carlton AFL Football Club); and CrownBet.com.au (AFL). For example, an advertisement from Centrebet.com.au featured a number of fan rituals, including images of St Kilda Football Club fans dressed in their team colours, having their faces painted in team colours, and fans out of their seats cheering for their team. These images were interspersed with betting imagery, including images of a man looking at the Centrebet.com.au website on his computer over a coffee. At the end of the advertisement the phrase *“Don’t Just Watch It”* was used to link fan behaviours to engagement in wagering. Similarly, the ‘It’s Your Call’ advertisement for Williamhill.com.au featured a young man making a decision about what to buy at a food truck at a sporting match, before deciding to purchase a meat pie. The advertisement included images of fans in the crowd dressed in AFL team colours, images of the centre bounce of the football, and images from other sporting events such as golf and American Football.

Celebrity endorsement was sometimes used to reinforce loyalty towards the sporting code and associated rituals. For example, former Australian Socceroos Goalkeeper Mark Schwarzer was used in a TAB.com.au campaign relating to sports wagering on the 2014 Soccer World Cup. Schwarzer is shown looking for information about the World Cup on the TAB.com.au website on his iPad, and is shown at a home cheering for the Australian national team. Wearing the Australian national colours of green and gold, and lying on a green and gold hammock, he is surrounded by male and female friends also wearing green and gold and cheering on the team. The match commentary in the background highlights both excitement, and nationalism: *“And the Socceroos are on the attack, let’s see what Australia can do here.”*

### Mateship and peer bonding

Advertisements were clearly targeted towards young men. This was evident from the actors and storylines within the advertisements. About half of the advertisements in this sample contained symbols and rituals that related to mateship (*n* = 41, 48 %). In these advertisements, gambling was portrayed as something that you did with friends, or in social settings. Most creatives featured groups of male friends engaging in gambling, with women mostly featuring in subordinate service roles in the advertisements. Male peer group activities depicted young men (in their mid to late 20s) watching sport together, socialising at the pub, or at social gatherings such as parties or barbeques. Visuals and story lines relating to mateship were backed up by narratives, with words such as *“mate”*, *“boys”* and *“lads”* commonly used within these advertisements.

Advertisements also linked mateship directly to gambling and a love of sport, embedding gambling in the social rituals associated with being a sports fan. The most prominent example of this was the Ladbrokes.com.au ‘AFL Season’ advertisement. The advertisement featured a group of friends engaging in a game of AFL in the ‘Ladbrokes Lounge’- a plush room featuring sporting memorabilia, trophies, couches and a snooker table. The ‘mates’, wearing jerseys with the Ladbrokes logo, finish the game and start gambling on AFL games together on their mobile phones, with the voice-over stating:

*“But no matter how much we all love to be those heroes, there comes a time to gather around the telly with your mates, and back the real ones. And whichever team you back, bet better with Ladbrokes.”*

This message was further reinforced with the positioning of the AFL brand logo at the end of the advertisement next to the Ladbrokes logo.

While television advertisements for betting and gambling are prohibited to associate gambling with alcohol [56], some advertisements appear to be set in pubs or bars. For example, in the TAB.com.au ‘How’s your Form?’ advertisement, a group of Caucasian young male adults were featured together in a sports bar. Likewise, Sportsbet.com.au’s ‘Gotta Love a Winner’ advertisement showed two young men betting against each other at a pub surrounded by their peers. This theme constructed gambling as a new social norm for groups of young men, alongside other ritualistic behaviours.

Wagering was also promoted as creating new and more extravagant social opportunities for social groups. For example, the creative for the Ladbrokes.com.au ‘Bet Anywhere, Shout Everywhere’ advertisement, showed a group of five male friends as they used their Ladbrokes Visa card to ‘shout’ each other lunch, flights, and holiday accommodation. The tagline for this advertisement was that with a Ladbrokes Visa card, mates could *“bet anywhere, shout everywhere”*. These advertisements suggested that winning (from gambling) could not only make the individual happy, but could also bring friends closer together. For example, in the TAB.com.au ‘Walk of Fame’ advertisement, the main actor and his friends are all depicted as happy, and are laughing along with the main actor’s win.

### Gender stereotypes and sexualised imagery

There was clear gender stereotyping in just under half of all advertisements (*n* = 34, 40 %). A quarter contained appeals that objectified women (*n* = 21, 25 %). The central actors within the advertisements were mostly Caucasian, heteronormative men, with the exception of one advertisement featuring African American actor Samuel L. Jackson. Men were the central actors within the advertisements with women primarily featured in secondary, service or subordinate roles. Women were also sexually objectified in advertisements, with advertisements often portraying male dominance or power over women. For example, an advertisement by Sportsbet.com.au (‘Boobs TVC’) described the bikini as *“man’s greatest invention”* while a man poked the breast of a woman in a bikini as she sunbathed by a pool. In the Betfair.com.au ‘Power to the Punter’ advertisement, a wealthy James Bond-type character in a suit played table tennis with a woman wearing a bikini. The voiceover states:

*“When you have power, you can do what you want. With whoever you want. Whenever you want. Wherever you want. In as many different ways as you want.”*

Women’s subservience to men was also evident in some advertisements. For example, in the TAB.com.au ‘How’s your Form’ advertisement, women featured in service roles behind the bar. In the Betfair.com.au ‘Power to the Punter 15 s TVC’ advertisement, a woman in a swimsuit and high heels, stood beside two men playing a game of Chinese checkers, while holding a platter of shellfish. In the background another woman sunbathes by the pool. The man bets on his mobile phone, with the narrative again drawing a link between gambling and power:

*"When you have power, the world is your oyster……. and your soft shell crab. Thank you Pam."*

Finally, women were portrayed in fantasy situations. For example, the Centrebet.com.au ‘Fire Up’ advertisement features Australian, female athlete Lauren Eagle and four other females dressed in short black dresses walking towards two men in a bar to the rock song ‘Black Betty’ by Spiderbait. As the women approach the bar, Eagle leans into the man and whispers *“Fire Up”*, at which point the man starts betting on his mobile phone.

There were two key stereotypes of men in wagering marketing. In the vast majority of advertisements, male stereotypes were focused on the average Australian male – the quintessential ‘average Aussie bloke’. Sports wagering was portrayed as a way to escape the ordinary by either: a) becoming more attractive to women (TAB.com.au ‘How’s Your Form’); b) gaining power and authority (Betfair.com.au ‘Power to the Punter’); and/or c) being able to afford a glamorous lifestyle (Ladbrokes.com.au ‘Bet Anywhere, Shout Everywhere’). For example, Sportsbet.com.au’s ‘Man Hands’ advertisement depicted the way in which ordinary men can escape the mundanity of everyday life. The advertisement describes the frustrations of the ‘average man’, and ways in which the gambling app could help to overcome daily struggles:

*“No more my man. Bet like a man, a man-handed man with the all new Sportsbet app for iPad. Sportsbet, bigger, better, betting.”*

Even celebrities and sports stars featured in advertisements were portrayed as the ‘average Aussie bloke’. For example, former Socceroos goalkeeper Mark Schwarzer was depicted as a patriotic sports fan, while former Australian cricketer Shane Warne was shown facing a series of challenges from Sportingbet.com.au. These included taking 50 paintballs, going a round with a female kickboxer, and facing his fear of spiders. In contrast, bookmakers were portrayed as powerful characters in advertisements. For example, in the TomWaterhouse.com.au ‘I know what punters want’ advertisement, Tom Waterhouse is dressed in a suit, and is shown directing his employees. Waterhouse is a symbol of power and wealth, and is far from the symbolic representation of the ‘average Aussie bloke’.

### Power, control, and social superiority

About one in five advertisements featured symbolism relating to power and control (*n* = 16, 19 %), and just under a third (*n* = 25, 29 %) contained appeals that linked gambling with social status and superiority. These concepts were utilised in many different ways throughout the advertisements. Most commonly ‘power’ and ‘control’ were used to suggest that an individual would have control over the way in which they gambled, and when and where they could gamble. For example, numerous promotions highlighted that wagering was always accessible via internet technologies, including an advertisement from Sportingbet.com.au (‘The World of Sportringbet-30 TVC’) that reminded gamblers they could *“bet anywhere, anytime”.* Wagering companies highlighted within advertisements that power over betting was being given back to gamblers via multiple betting options, choices, services, and ultimately winnings. For example, the Betfair.com.au’s ‘Cash Out’ advertisement implied that the outcome of betting was something that the gambler could control via its ‘cash out’ service:

*“It used to be that the fate of your bet was left to the Gods. But thanks to Betfair cash out, we put the fate in your hands. Cash out at any stage of the tournament, and control the fate of your bet.”*

Similarly, Sportsbet.com.au’s ‘Freedom’ features a group of young men at a barbeque. The campaign highlights the young man gaining a sense of freedom over social situations via the ‘in game cash out’ function on the Sportsbet.com.au mobile app. The storyline describes how the young man can feel an *“overwhelming sense of freedom”* and that gamblers can *“unlock your potential”* with the Sportsbet.com.au app. He is depicted breaking accepted social etiquettes, including leaving the toilet seat up, taking his trousers off and walking around the barbeque in his underpants, and placing a drink on the table right next to a coaster.

Concepts of power and control that resulted from wagering were also linked to concepts of social status. The key message within these advertisements was that the financial success from wagering would lead to affluent lifestyles, and control over life choices. For example Williamhill.com.au’s ‘2015 NRL Finals’ advertisement, depicts a wealthy, suited man, accompanied by the tagline, *“Own the moment this NRL Finals Series with William Hill.”*

### Adventure, thrill, and risk

While many advertisements used messages about control, some advertisements used appeal strategies related to adventure, thrill, and risk (*n* = 23, 27 %). Narratives specifically relating to risk were featured in some advertisements. For example, a TomWaterhouse.com.au advertisement featured the phrases *“we don’t get rewards, without the risks”*, and describes an athlete who pushes the limits so that he is able to feel stronger. The implication is that gamblers need to take risks to reap higher rewards. Narratives relating to thrill were also featured in some advertisements. Some specifically portrayed gambling as a way of increasing the thrills associated with the sporting experience. For example UBET.com.au’s advertisement for the first State of Origin National Rugby League game ‘Origin 1 first try special’ depicts Queensland and New South Wales fans up out of their seats cheering for their team. The accompanying narration states:

*“Wanna up the thrill at this year's State of Origin? You bet!! When you place a bet on first try scorer, UBET will pay out if your player scores first or second. Place your bets at your local TAB or download the UBET app. For the thrill of it - UBET”.*

### Winning

Winning was symbolised in about a third of all advertisements (*n* = 30, 35 %). Winning was predominantly linked to themes of personal and group triumph and the social success resulting from monetary gains. Winning money was featured in a number of advertisements. In Sportsbet.com.au’s ‘Just Awesome Odds’ advertisement a young man is shown dancing with a huge bag of money after a gambling win. As he hugs the bag of money, notes fly out and saturate the screen. Advertisements also depicted individuals withdrawing money and collecting winnings. For example, TAB.com.au’s ‘Synchronised Winners’ advertisement portrays three young male winners, who dance their way to the cashier counter to collect their money. In Sportsbet.com.au’s ‘Big Shout’ and ‘Little Shout’ advertisements, young men were shown winning at the pub, using the Sportsbet.com.au cash card to withdraw winnings from Automated Teller Machines, and then ‘shouting’ their mates. In these commercials, winning was also linked to social status and popularity with groups of friends.

## Discussion and implictions

This study aimed to explore the symbols, rituals and meanings contained in sports wagering advertisements in Australia. Researchers have shown that symbolic consumption (and identity creation) were important advertising strategies in the promotion of unhealthy commodity products such as alcohol and tobacco [39–43]. Our study suggests that the sports wagering industry may be operating from the same playbook, using multiple symbolic consumption strategies to influence the social acceptance of sports wagering, thus shaping the cultural meanings that specific audience segments have about the relationship between gambling and sport [27, 28]. The most overt strategy identified in our analysis was the use of creatives that sought to embed sports wagering directly with sports rituals and practices. Product alignment with sports is not a new concept, and is a traditionally favoured technique utilised by other potentially harmful commodity industries, such as tobacco and alcohol [26, 50, 59–61]. However, we would also argue that the alignment between wagering and sport in marketing strategies is amplified because sport is, in essence, the product for wagering companies. Rather than just an ‘add on’ to existing sporting practices, wagering advertising appears to be embedding gambling as a socially and culturally accepted activity that is *central* to sporting rituals and practices, being a loyal sports fan, and in some cases a patriotic Australian. This potentially makes sports wagering advertising during or aligned with sport an exceptionally influential form of promotion.

Sports wagering advertising creatives also sought to align gambling with peer-based social activities, not only as they related to sport, but also in other social settings. Many advertisements featured groups of young adult males. Sports wagering was depicted as an activity that facilitated social cohesion, mateship, and social opportunities. The depiction of ‘the male group’ appears in Strate’s [44] analysis of alcohol advertisements; he notes that such a depiction *“plays on the common misconception that drinking, when it is done socially and publically, cannot be harmful”* [p 88]. Similarly, our interpretation of advertisements suggested that wagering on sport was an activity that had numerous social and financial benefits for young men. The interplay between, and the symbolic representation of, fan loyalty behaviour, mateship and masculinity serve to associate an already valued and established ritual for young male sports fans with that of gambling. Key to these representations is the depiction of gambling in social situations, overlaid with symbols of mateship. We would argue that this distinguishes sports wagering as somehow separate from ‘hard’ forms of gambling (Electronic Gambling Machines), and sports bettors as in some way (positively) dichotomised from that of the ‘problem gambler’. These representations lend themselves to social acceptability and allow young men to embody the symbolically suggested benefits of sports wagering.

The current study also highlights the use of gendered messages within wagering advertisements. Our interpretation was that advertising utilised symbols associated with the ‘average Aussie bloke’, and overwhelmingly featured young men as the central actors within creatives. Similar strategies have been observed in alcohol marketing strategies, which align symbolic representations of a ‘holy trinity’ between sport, masculinity (and ‘the lads’) and alcohol consumption [44, 62–64]. Women were predominantly featured in secondary roles within advertisements, and were often objectified in swimwear or in fantasy situations. Again there are similarities with alcohol advertising strategies whereby women were deviod of their personality, intelligence and power, and portrayed (and viewed) as objects of sexual desire [65]. In our analysis, humour was at times used to excuse these messages, playing upon the notion that such depictions and messages are ‘acceptable’, when communicated in a particular format. In this way, the sports wagering industry are able to circumvent accountability, if their advertising is layered with humour and comedy. It is important to note that where there have been community complaints to advertising standards bodies about the portrayal of women in gambling advertising, these complaints have not been successful. For example, in 2012 a complaint to the Advertising Standards Bureau (ASB) claimed that the Centrebet.com.au advertisement featuring Lauren Eagle breached sections of the advertising Code of Ethics that related to gender vilification, sex, and health and safety. This was dismissed by the ASB. It is interesting to note that the complaint specifically compared the discrepancy between acceptable standards in alcohol and gambling advertising:

*"If this was an alcohol ad, it would not have been permitted to air. Gambling, which has similar potential to cause harm on personal and social levels, is depicted as attractive and leading to sexual success.”* [66]

While there is widespread concern about the inadequacies of the current voluntary alcohol advertising self-regulatory system, this ruling suggests that advertisements for gambling are addressed with an even lighter touch.

## Conclusions

While some research suggests that these marketing tactics are having an impact on young men’s consumption of wagering products as part of their existing sporting rituals within peer groups [24], further research is needed to investigate how these processes of ‘*symbolic consumption’* are occurring, and how marketing may contribute to a new set of individual and peer group identities related to gambling on sport [35, 36]. This includes seeking to understand whether different audience segments are co-creating, developing or attaching new meanings to wagering products [34]. Researchers will be able to draw on previous research particularly from tobacco and alcohol [29–33] when developing studies that seek to understand the roles a range of advertising strategies (and broader strategies used by the wagering industry such as sponsorship, and the offering of inducements and incentives to gamble) may play in shaping gambling beliefs and consumption intentions. Monitoring how campaign tactics change over time, and more detailed investigations of the thematic similarities and differences in the creative strategies for different wagering brands, will also be important in understanding the marketing strategies of the sports wagering industry.

Given the identified harms from gambling, and the ways in which gambling marketing and opportunities to gamble have proliferated, policy makers and public health practitioners need to develop effective regulatory mechanisms to respond to the practices observed within gambling marketing strategies.

## Abbreviations

AFL: Australian Football League
ASB: Advertising Standards Bureau
